# Medical and Philosophical News

**Published:** 1781-02

**Authors:** 


The section of the symphysis pubis has lately been performed with success in Spain, by Signor Canivell, surgeon major to the Naval Hospital at Cadiz. The patient, a lady aged forty-two years, was taken in labour of her first child on the 6th of August last. The labour pains continued during twenty-four hours, but the head of the fœtus being prevented from advancing by the narrowness and distortion of the pelvis, and the patient being much reduced, her accoucheur thought the operation adviseable. It was accordingly performed without wounding the integuments. The incision was begun above the clitoris, and the operator was careful to injure the meatus urinarius as little as possible. As soon as the separation was effected, a crepitus was heard, and in a few minutes the patient was delivered of a live child. The fever soon went off, and on the 38th day she got out of bed. The only inconvenience she feels at present, is a slight incontinence of urine.

